# *Helicobacter pylori* Eradication in Idiopathic Thrombocytopenic Purpura: A Meta-Analysis of Randomized Trials

**DOI:** 10.1155/2018/6090878

**Published:** 2018-10-09

**Authors:** Bum Jun Kim, Hyeong Su Kim, Hyun Joo Jang, Jung Han Kim

**Affiliations:** ^1^Division of Hemato-Oncology, Department of Internal Medicine, Kangnam Sacred-Heart Hospital, Hallym University Medical Center, Hallym University College of Medicine, Seoul, Republic of Korea; ^2^Department of Internal Medicine, National Army Capital Hospital, The Armed Forces Medical Command, Sungnam, Gyeonggi-do, Republic of Korea; ^3^Division of Gastroenterology, Department of Internal Medicine, Dongtan Sacred-Heart Hospital, Hallym University Medical Center, Hallym University College of Medicine, Hwasung, Gyeonggi-do, Republic of Korea

## Abstract

**Objective:**

Several recent reviews of published studies have shown that the eradication of *H. pylori* infection in patients with ITP improved thrombocytopenia in about half of the cases. However, most included studies were observational case series. We performed the first meta-analysis of randomized trials to gain a better insight into the effect of *H. pylori* eradication in ITP patients.

**Methods:**

A systematic computerized search of the electronic databases including PubMed, EMBASE, Google Scholar, and Cochrane Library (up to December 2017) was conducted.

**Results:**

From six studies, a total of 241 patients (125 in eradication group and 116 in control group) were included in the meta-analysis. Patients in the eradication group showed significantly higher overall platelet response rate than those in the control group (odds ratio = 1.93, 95% confidence interval: 1.01–3.71, *P* = 0.05). In the subgroup analysis, however, children in the eradication group failed to show statistically better response rate than those in the noneradication group (odds ratio = 1.80, 95% confidence interval: 0.88–3.65, *P* = 0.11).

**Conclusions:**

This meta-analysis indicates that *H. pylori* eradication has a significant therapeutic effect in patients with ITP. Considering the intrinsic limits in the design and sample size of the included studies, however, large randomized controlled trials are warranted to validate the therapeutic impact of *H. pylori* eradication in adults as well as children with ITP.

## 1. Introduction

Idiopathic or immune thrombocytopenic purpura (ITP) is an autoimmune-mediated acquired bleeding disorder of children as well as adults. It is characterized by the destruction of host platelet caused by anti-platelet antibodies [1]. However, the mechanisms that trigger the development of platelet auto-antibodies remain poorly understood. Persistent thrombocytopenia for more than 6 or 12 months defines the chronic form of ITP [2, 3]. ITP is typically a diagnosis of exclusion, made by clinicians after ruling out other possible etiologies. ITP can be a primary disease or secondary to a variety of etiologies including bacterial or viral infection, autoimmune disease, or neoplasm [1–3].

*Helicobacter pylori (H. pylori)* is the most common microbial pathogen that colonizes in the mucosal layer of the stomach. It is causally associated with a variety of gastrointestinal disorders including chronic gastritis, gastric mucosal atrophy, peptic ulcer, gastric mucosa-associated lymphoid tissue lymphoma, and gastric adenocarcinoma [4, 5]. A pathophysiologic link between ITP and *H. pylori* infection was initially proposed in 1998 by Gasbarrini et al. who reported a significant increase of platelet count after bacterial eradication in 8 of 11 ITP patients infected with *H. pylori* [6]. Although the pathogenesis of *H. pylori*-associated ITP is still uncertain, several studies have suggested that *H. pylori* virulence factor, cytotoxin-associated gene A (CagA), stimulates the development of anti-CagA antibodies (Abs) that cross-react with platelet surface antigens (Ags), resulting in thrombocytopenia [7–9]. Many studies have reported that *H. pylori* eradication led to an increase of platelet counts and even a regression of ITP [10–18]. As other studies have failed to demonstrate the beneficial effect of bacterial eradication in ITP [19–21], however, there is a debate as to whether the eradication of *H. pylori* in chronic ITP is effective in increasing platelet counts or not.

Several recent reviews of previously published studies have shown that the eradication of *H. pylori* infection in patients with chronic ITP improved thrombocytopenia in about half of the cases [22–24]. The metaregression model revealed that the success of bacterium eradication was highly significant as an explanatory variable for increase of platelet count [22]. However, most studies included were observational case series [22–24], which might subject the results to possible bias. In addition, several recent randomized trials in adults or children showed the inconsistent effect of bacterium eradication on platelet recovery in ITP patients infected with *H. pylori* [16–21]. Therefore, we performed this meta-analysis of randomized trials to gain a better insight into the effect of *H. pylori* eradication in ITP patients.

## 2. Materials and Methods

### 2.1. Publication Searching Strategy

The current study was conducted according to the preferred reporting items for systematic reviews and meta-analyses (PRISMA) guidelines [25, 26]. A systematic computerized search of the electronic databases including PubMed, EMBASE, Google Scholar, and Cochrane Library (up to December 2017) was performed. The search used the following keywords variably combined: “*Helicobacter pylori*,” “*H. pylori*,” “thrombocytopenia,” “idiopathic thrombocytopenic purpura,” “immune thrombocytopenic purpura,” and “ITP.” The related article function in PubMed was used to identify all relevant articles. In addition, the bibliographic references of all retrieved studies and reviews were evaluated for additional eligible articles.

### 2.2. Inclusion Criteria

We only included randomized controlled trials in this meta-analysis. Retrospective or observational case control studies were excluded. Eligible studies should meet the following inclusion criteria: (i) patients with a diagnosis of chronic ITP according to the American Society of Hematology (ASH) guidelines [2]; (ii) *H. pylori* infection documented by reliable tests such as ^13^C-urea breath test (UBT), serologic test for antibody to *H. pylori*, stool antigen test, or histology of gastric mucosal biopsies; (iii) randomization of ITP patients infected with *H. pylori* to either bacterial eradication or noneradication; (iv) providing treatment outcomes (platelet counts or response rate) of these two groups. Reports published only in abstract form were not considered eligible.

### 2.3. Data Extraction

Two reviewers (BJK and HJJ) independently screened relevant studies and extracted the data from each eligible study. If these two authors did not agree, the other investigator (JHK) was consulted to resolve the disagreement through discussion.

The following data were extracted from the included studies: the first author, year of publication, country, number of patients, demographics (age, gender), detecting methods for *H. pylori* infection, duration of ITP, treatment, platelet counts (before and after treatment) or treatment outcomes, and relapse rate.

### 2.4. Quality Assessment

The methodological quality of the randomized trials was scored using the Jadad five-item scale, taking into account randomization, double-blinding process, and withdrawals [27]. The final score ranged from 0 to 5, with low-quality studies having a score ≤ 2 and high-quality studies having a score of ≥3.

### 2.5. Statistical Analyses

We chose to record the overall response rate (ORR) as primary assessment criteria. The odds ratios (ORs) and 95% confidence intervals (CIs) for ORR were calculated indirectly from original articles. The effect size of ORR was pooled through OR and its 95% CI. The heterogeneity across studies was estimated by the *Q* statistics and *I*^2^ inconsistency test. The fixed-effect model (Mantel–Haenszel method) was used for pooling homogeneous outcomes (*P* ≥ 0.1 and *I*^2^ ≤ 50%), and the random-effect model (DerSimonian–Laird method) was selected if significant heterogeneity was observed (*P* < 0.1 and *I*^2^ > 50%).

The RevMan version 5.3 was used to combine the data. The plots show a summary estimate of the results from all the studies combined. The size of the squares represents the estimate from each study, reflecting the statistical “weight” of the study. Outcomes are provided as forest plots with diamonds representing the estimate of the pooled effect and the width of diamond implying its precision. The line of no effect is number one for binary outcomes, which depicts statistical significance if not crossed by the diamond [28]. The OR > 1.0 implies better response for patients receiving the eradication treatment of *H. pylori* infection.

The possibility of publication bias was assessed with a visual inspection of the graphical funnel plot [29]. The statistical methods for detecting funnel plot asymmetry were the rank correlation tests of Begg and Mazumdar and Egger's regression asymmetry test [29, 30]. Statistical significance was considered for a *P* value of less than 0.05 for the summary estimate of OR and publication biases.

## 3. Results

### 3.1. Results of Search

**Figure 1:**
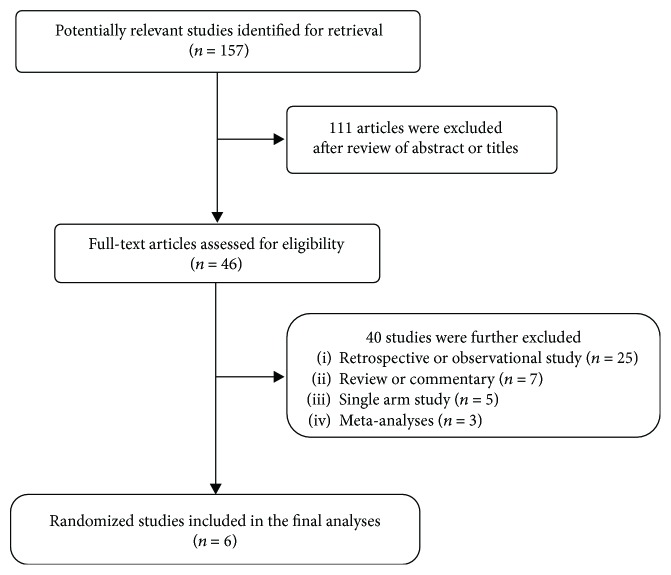
Flow diagram of search process.

A total of 157 potentially relevant articles were initially found, but 111 of them were excluded after careful screening of the titles and abstracts. We retrieved 46 articles for full-text evaluation and further excluded 40 by the inclusion criteria. Finally, 6 studies were included in the meta-analysis [16–21].  Figure 1 shows the search flow diagram of this meta-analysis.

### 3.2. Characteristics of the Included Studies

Table 1 summarizes the main characteristics and treatment outcomes of the six studies. Four studies were conducted in children [18–21] and the remaining 2 in adults [16, 17]. The most common detection method for *H. pylori* infection was UBT [16, 18–21]. The prevalence of *H. pylori* infection ranged from 25.9% [21] to 73.9% [20]. Bacterium eradication consisted of standard triple therapy including clarithromycin, amoxicillin, and proton-pump inhibitor (PPI) (omeprazole or lansoprazole) for 7–14 days. Except for one study [16], patients in the control arms were usually treated with corticosteroid (prednisone or prednisolone) or PPI alone.

### 3.3. Platelet Response to Treatment

In most studies, complete response (CR) was defined as the achievement of a platelet count more than 150 × 10^9^/L [16, 18–21]. However, the threshold for partial response (PR) varied among studies although platelet count with a net increase of greater than 30 × 10^9^/L was most commonly adopted [17, 18, 20, 21] (Table 1). We defined the ORR by adding CR rate and PR rate. The ORR varied from 14.3% [19] to 88.2% [18] in the eradication arms and from 0% [16] to 88.1% [18] in the noneradication arms.

### 3.4. Quality of the Included Studies

The Jadad scores for the 6 randomized studies was 2 or less. The studies announced that patients were randomly assigned by concealed allocation, but no more information on the method to generate the sequence of randomization was available.

### 3.5. Therapeutic Effect of *H. pylori* Eradication: Meta-Analysis

**Figure 2:**
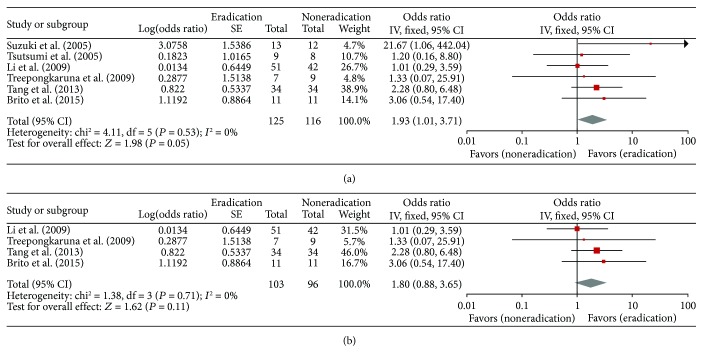
Forest plots of odds ratios for overall platelet response rates in all patients (a) and in children (b).

From the six studies, a total of 241 patients (125 in the eradication group and 116 in the control group) were included in the meta-analysis of ORs for ORR. The fixed-effect model was selected because there was no significant heterogeneity among studies (*X*^2^ = 4.11, *P* = 0.53, *I*^2^ = 0%). Patients in the eradication group showed significantly higher ORR than those in the control group (OR = 1.93, 95% CI: 1.01–3.71, *P* = 0.05) (Figure 2(a)).

In the subgroup analysis, children in the eradication group failed to show statistically higher ORR than those in the noneradication group (OR = 1.80, 95% CI: 0.88–3.65, *P* = 0.11) (Figure 2(b)). There was no significant heterogeneity among studies (*X*^2^ = 1.38, *P* = 0.71, *I*^2^ = 0%), and the fixed-effect model was used for pooling the data.

### 3.6. Publication Bias

**Figure 3:**
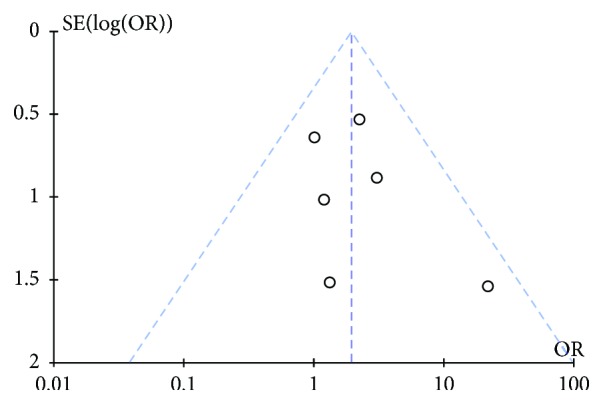
Funnel plot for publication bias regarding overall response rates.

Begg's funnel plot and Egger's test indicated no evidence of substantial publication bias for ORR (Begg's *P* = 0.452, Egger's *P* = 0.465) (Figure 3).

## 4. Discussion

Despite the findings indicating that *H. pylori* infection plays an etiological role in ITP, several randomized trials to date have shown the inconsistent results in the effect of bacterium eradication. In this meta-analysis of 6 randomized trials, we evaluated the therapeutic effect of *H. pylori* eradication in patients with ITP. Our results indicate that bacterium eradication has a significant impact on platelet recovery in ITP.

ITP is considered an organ-specific autoimmune disease. It is mediated by anti-platelet Abs that bind to host platelets and megakaryocytes, accelerating platelet destruction by the reticuloendothelial system [31]. The auto-Abs primarily target platelet surface glycoproteins such as GP IIb/IIIa and GP Ib. Although the triggering factors for ITP are obscure, bacterial or viral infections are known to be associated with the development of ITP, indicating that infectious agents may play a critical role in the pathogenesis of a particular subset of ITP [32].

Since the first report in 1998 [6], the accumulating data have indicated that *H. pylori* may contribute to the pathogenesis of chronic ITP [7, 9, 31–34]. In addition, numerous clinical studies have reported that *H. pylori* eradication resulted in an increase of platelet counts in ITP [10–18]. Various mechanisms have been proposed for the role of *H. pylori* in chronic ITP: the production of Ag-specific Abs that are cross-reactive with platelet surface glycoproteins (GP IIb/IIIa or GP Ib) due to molecular mimicry increased plasmacytoid dendritic cell numbers and variable host immune response to virulence factors such as vacuolating-associated cytotoxin gene A (VacA) and CagA [7, 9, 33, 34]. A recent study in China reported that the Fc*γ*RIIB expression on circulating monocytes is downregulated in *H. pylori*-infected ITP patients [35]. Therefore, *H. pylori* infection may play an important role in the development of ITP by activating the Fc*γ* receptor of monocytes/macrophages through downregulation of the inhibitory receptor Fc*γ*RIIB. Despite mounting evidence of the association of *H. pylori* and ITP, published studies are inconclusive due to the scarcity of controlled clinical trials to date and some reports presenting conflicting results [16–21]. Moreover, empirical treatment of *H. pylori* in children with ITP remains controversial [36, 37]. Therefore, validating the therapeutic effects of *H. pylori* eradication has a critical impact on clinical diagnosis and treatment of ITP.

In the previous systemic reviews, the pooled prevalence of *H. pylori* infection in ITP ranged from 58% [38] to 65% [23]. The first meta-analysis by Franchini et al. reviewed 17 studies with 788 ITP patients, including 494 infected with *H. pylori* [22]. There was a statistically significant difference in the increase of platelet count in patients for whom *H. pylori* eradication was successful, compared with untreated patients (weighted mean difference (WMD) of 40.77 × 10^9^/L, 95% CI: 20.92–60.63) and those who failed eradication (WMD of 52.16 × 10^9^/L, 95% CI: 27.79–64.91). Another systemic review with a meta-analysis of 1555 patients with ITP from 25 studies reported the weighted mean CR (platelet count more than 100 × 10^9^/L) of 42.7% and ORR (platelet count ≥ 30 × 10^9^/L and at least doubling of basal platelet count) of 50.3% after successful eradication of *H. pylori* [23]. In addition, the most recent systemic review by Frydman et al. confirmed that eradication treatment in *H. pylori*-positive adult ITP patients resulted in about a 50% CR rate [24]. These findings indicate the beneficial effect of bacterium eradication in ITP patients infected with *H. pylori*. Except for one [16], however, almost all articles included in those reviews were retrospective or observational studies [22–24]. Thereafter, five more randomized trials were conducted in adult or children ITP patients [17–21]. While three studies observed a significant difference in the platelet response between the eradication group and noneradication group [16, 20, 21], there was no beneficial effect of *H. pylori* eradication in the other studies [17–19].

In this meta-analysis of 6 randomized trials [16–21], the prevalence of *H. pylori* infection ranged from 30% [21] to 74% [20]. Patients who received bacterium eradication therapy showed significantly higher ORR than those in the control group (OR = 1.93, 95% CI: 1.01–3.71, *P* = 0.05). This result suggests that *H. pylori* eradication in patients with ITP is effective in increasing platelet count. The recent ASH guidelines have recommended the examination and treatment of *H. pylori* infection in adult patients with ITP [2]. However, routine testing for *H. pylori* in children is not recommended. The prevalence of *H. pylori* infection is much lower in children than in adults with chronic ITP, suggesting that *H. pylori* play only a minor role in the development of childhood ITP [39]. Platelet responses after *H. pylori* eradication in children were highly variable and inconsistent among studies [18–21, 39–41]. In the subgroup analysis of the current study, children in the eradication group failed to reach statistical significance to show higher ORR than those in the control group (OR = 1.80, 95% CI: 0.88–3.65, *P* = 0.11). However, the subgroup analysis included only 4 studies with a small number of patients. Therefore, this finding seems not sufficient to determine the therapeutic efficacy of *H. pylori* eradication in childhood ITP. Larger randomized trials are necessary to confirm the beneficial role of *H. pylori* eradication in childhood ITP.

A number of questions regarding the eradication of *H. pylori* infection in ITP still remain to be resolved, including the difference of efficacy among countries, factors predicting the platelet response, and mechanisms responsible for therapeutic effect associated with *H. pylori* eradication [39]. There was a great variability in the platelet responses of *H. pylori* eradication between Western and Eastern series [23]. Studies from Japan tended toward better response rates (28%–100%) than those from USA, Spain, and Mexico (<20%). The reason for such variability among countries is not clear, but geographic differences in the epidemic *H. pylori* strains may, at least in part, account for the difference in the platelet responses. The frequency of CagA-positive strains varies among geographic locations. Eastern Asian *H. pylori* strains have been found as more pathogenic, correlating with an increased development of gastric adenocarcinoma in *H. pylori*-positive patients in East Asia [42, 43]. The majority of *H. pylori* strains detected in East Asia express CagA, whereas the proportion of CagA-positive *H. pylori* strains in Western countries was much lower [44]. Immune response to CagA protein may be associated with improved platelet count after *H. pylori* eradication in patients with ITP [7–9]. Takahashi et al. observed that the level of anti-CagA Ab in the platelet eluates declined in three patients showing CR after eradication [7]. Kodama et al. reported that reduction in the anti-CagA antibody titer after eradication therapy was significantly greater in responders than in nonresponders [9]. These findings suggest that anti-CagA Ab titer may be a biomarker to determine who is indicated for bacterium eradication among patients with ITP.

Our study has several inherent limitations that need to be discussed. First, this meta-analysis included a limited number of studies with a small sample size. Moreover, there were only two trials conducted in adults with ITP. Second, five studies were carried out in Asia and the remaining trial in South Africa. So the results might not be transferred to Caucasian populations. Third, the studies showed heterogeneity in the detection methods of *H. pylori* infection. Especially one study [17] adopted the serologic test which showed inferior sensitivity and specificity compared with other diagnostic methods [45]. Fourth, most studies had a low quality with Jadad score ≤ 2. Fifth, because of the paucity of data, we could not evaluate the difference in the increase of platelet count between the eradication group and control group. Finally, although the definition of CR was similar among studies, the threshold for PR varied a little.

In conclusion, our meta-analysis indicates that *H. pylori* eradication has a significant therapeutic effect in patients with ITP. This result suggests that the detection and eradication of *H. pylori* infection need to be considered in patients with chronic ITP. Taking into account the intrinsic limits in the design and sample size of the included studies, however, large randomized controlled trials are warranted to validate the therapeutic impact of *H. pylori* eradication in adults as well as children with chronic ITP.

## Figures and Tables

**Table 1:** Summary of the six included studies.

First author (year) Country [ref]	Number of ITP pts	Detection of Hp infection	Number of Hp (+) pts	Randomization	Number of pts	M/F	Mean age (yr) (SD or range)	Duration of ITP (yr)	Platelet at enrollment (×10^9^/L)	Platelet after 6 mo of Tx (×10^9^/L)	Response^‡^ (CR + PR)	Relapse at 1 year	Jadad score
Suzuki (2005) Japan [16]	36	UBT or histology	25 (69.4%)	Eradication	13	5/8	57.4 ± 15.0	5.8 ± 7.2	54.7 ± 26.9	114.5 ± 90.5	6 (46.2%)	NA	2
				Noneradication (observation)	12	5/7	56.2 ± 7.8	4.6 ± 5.2	48.4 ± 22.1	48.1 ± 26.0	0 (0%)	NA	

Tsutsumi (2005) Japan [17]	25	Anti-Hp antibody	17 (68%)	Eradication	9	2/7	60.3	NA	NA	NA	6 (66.7%)	2 (33.3%)	2
				Noneradication (PPI)	8	3/5	63.3	NA	NA	NA	5 (62.5%)	2 (40%)	

Li (2009) China [18]	NA	UBT	93	Eradication + PD	51	27/24	6.7 ± 2.4	NA	NA	NA	45 (88.2%)	11 (21.6%)	1
				Noneradication (PD)	42	22/20	5.8 ± 2.7	NA	NA	NA	37 (88.1%)	17 (40.5%)	

Treepongkaruna (2009) Thailand [19]	55	UBT	16 (29.1%)	Eradication + PD	7	3/4	11.0	3.4 (1.7–6.9)	23.0 (3.0–84.0)	NA	1 (14.3%)	NA	2
				Noneradication (PD)	9(8)^∗^	4/5	10.8	5.1 (1.2–9.5)	34.0 (3.0–86.0)	NA	1 (12.5%)	NA	

Tang (2013) China [20]	92	UBT	68 (73.9%)	Eradication ± PD	34	NA	NA (child)	NA	14.8 ± 0.4	160.4 ± 1.0	26 (76.5%)	NA	2
				Noneradication (±PD)	34	NA	NA (child)	NA	15.1 ± 0.3	80.6 ± 1.1	20 (58.8%)	NA	

Brito (2015) Brazil [21]	85	UBT or stool Ag test	22 (25.9%)	Eradication ± PD	11	6/5	12.7 (4.9–17.5)	5 (1–8)	35.0 (1–145)	128 ± 73	7 (63.6%)	NA	2
				Noneradication (±PD)	11	5/6	10.5 (5.8–17.7)	3 (0.7–11)	47 (8–139)	63 ± 44	4 (36.4%)	NA	

ITP: idiopathic or immune thrombocytopenia purpura; Hp: *Helicobacter pylori*; UBT: ^13^C-urea breath test; pts: patients; yr: years; mo: months; SD: standard deviation; PPI: proton-pump inhibitor, PD: prednisone or prednisolone; Tx: treatment; CR: complete platelet response; PR: partial platelet response; NA: not available. ^∗^One patient was withdrawn due to massive gastrointestinal bleeding, requiring high-dose prednisolone. ^‡^Overall response criteria: Suzuki: platelet count increased by more than 50 × 10^9^/L 6 months after eradication therapy; Tsutsumi: platelet count with a net increase greater than 30 × 10^9^/L or a 50% increase in platelet count with a net increase of 10 × 10^9^/L but less than 30 × 10^9^/L; Li: platelet count with a net increase greater than 30 × 10^9^/L; Treepongkaruna: platelet count more than 100 × 10^9^/L sustaining for at least 3 months; Tang: platelet count more than 50 × 10^9^/L or platelet count with a net increase greater than 30 × 10^9^/L; Brito: platelet increase greater than 20–30 × 10^9^/L.
